# African Bush Viper Envenomation: A Case Report

**DOI:** 10.7759/cureus.28040

**Published:** 2022-08-15

**Authors:** Nicholas S Imperato, Alexandra M Amaducci, Benjamin N Abo, Andrew L Koons, Derek J Fikse, Kenneth D Katz

**Affiliations:** 1 Emergency and Hospital Medicine, University of South Florida Morsani College of Medicine/Lehigh Valley Health Network Campus, Allentown, USA; 2 Emergency Medicine, Florida State University College of Medicine, Tallahassee, USA; 3 Emergency Medical Services, Miami-Dade Fire Rescue Venom Response Unit, Doral, USA

**Keywords:** african bush viper, antivenom, venomous, snake-bite, atheris squamigera

## Abstract

*Atheris squamigera* envenomation is an infrequently documented occurrence in the United States (US). Cases of envenomation may induce severe coagulopathies, renal failure, and potentially life-threatening hemorrhage. Currently, there are no antivenoms specific to the *Atheris* genus, but there have been documented cases of the use of antivenoms for other species. A 26-year-old man presented to the emergency department (ED) complaining of swelling and discomfort in his left foot after being bitten by an *Atheris squamigera *that he kept as a pet.After performing a physical exam, it was noted that the patient’s envenomation was likely mild. Throughout his hospital stay, he developed lab abnormalities, most notably an elevated D-dimer and low fibrinogen. His clinical symptoms improved after a short stay, and he did not require antivenom treatment. This case highlights a rare, but potentially life-threatening envenomation that may be encountered in the US due to the continued practice of exotic pet ownership and sales. Moreover, procurement of antivenom for non-native species poses a unique challenge to US physicians responsible for treating these patients.

## Introduction

*Atheris squamigera* envenomations in humans are rarely reported in the United States (US) medical literature. *Atheris squamigera*, more commonly known as Green Bush Vipers, Variable Bush Vipers, or African Bush Vipers, are a viper species normally found in the rainforest regions of sub-Saharan Africa [1]. *Atheris squamigera* envenomation can cause severe coagulopathy, pain, edema, renal failure, and on rare occasion, life-threatening hemorrhage [1]. Severe envenomations from *Atheris squamigera* bites require antivenom, but there are no species-specific antivenoms readily available in the US. An African Bush Viper’s venom acts through various disintegrins and snake venom metalloproteinases (SVMPs), which inhibit platelet aggregation and induce skin damage via degradation of dermal-epidermal junctions, respectively [2].

This article was previously presented as an abstract at the 2022 American College of Medical Toxicology Annual Scientific Meeting on March 12, 2022, and the 2022 Pennsylvania College of Emergency Physicians Scientific Assembly on April 1, 2022.

## Case presentation

**Figure 1:**
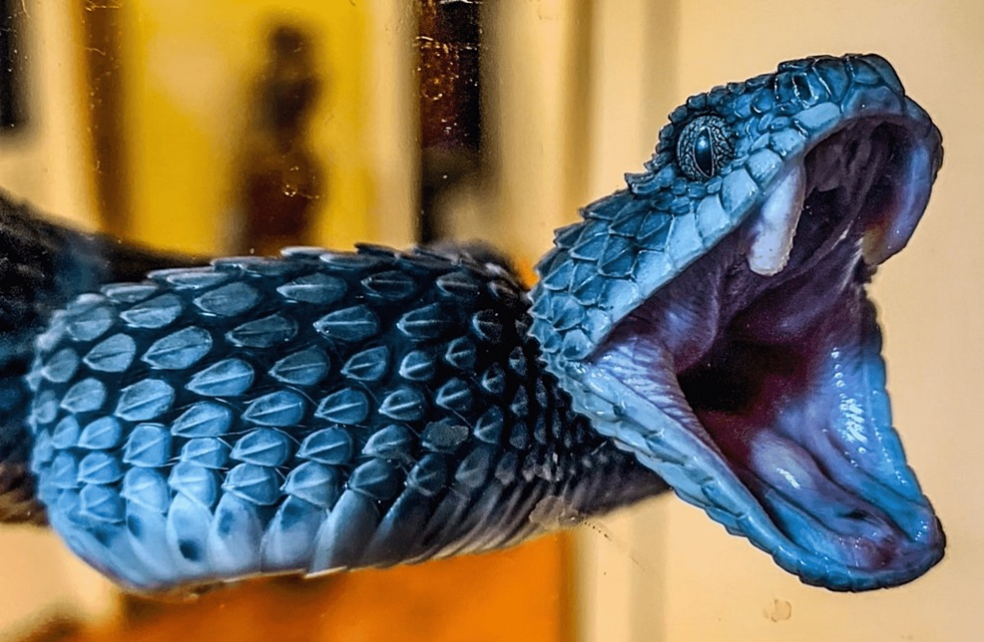
Atheris squamigera, more commonly known as the African Bush Viper. Photo credit: Khara Geder-Smith, Ngala Wildlife Preserve (reproduced with permission)

**Figure 2:**
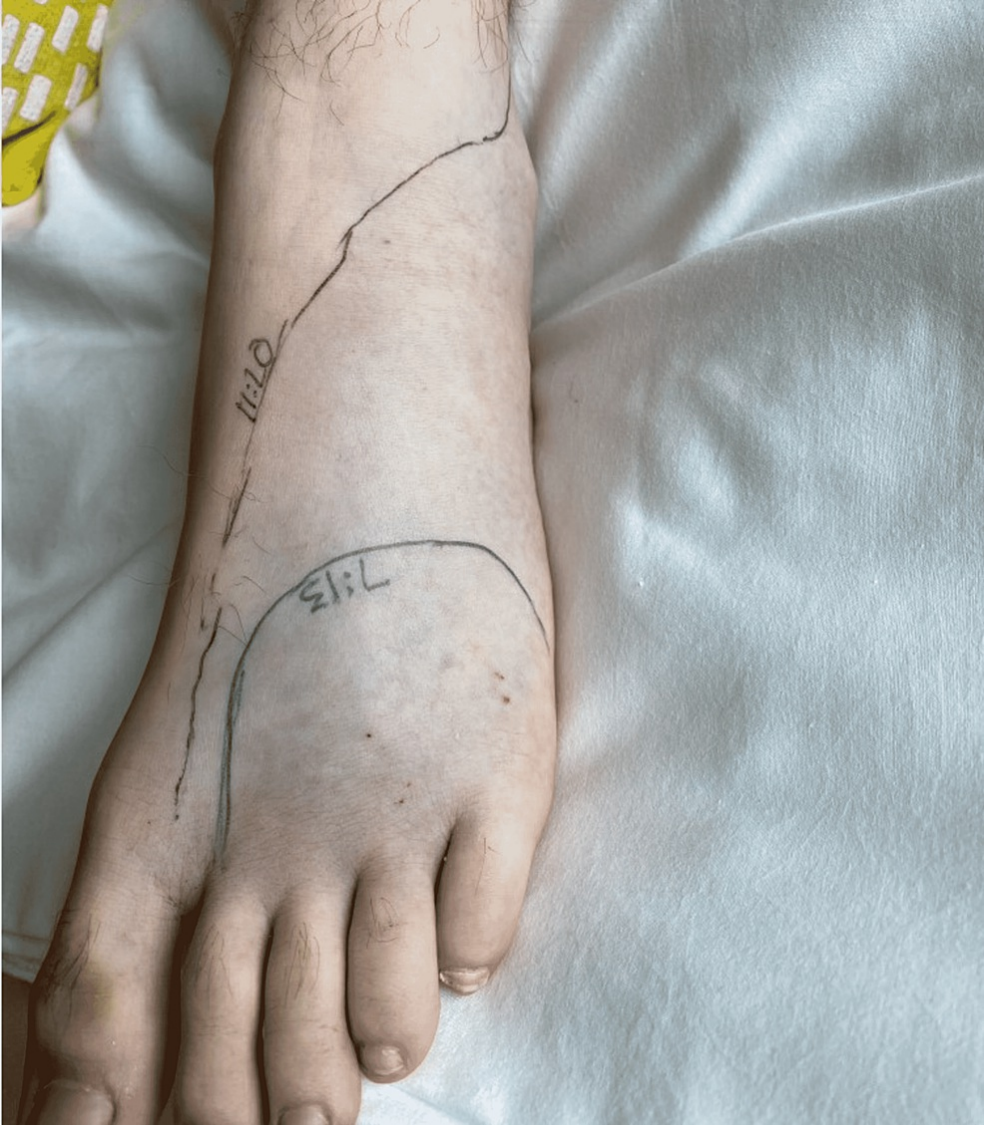
Lower extremity snake bite. Patient was bitten twice by his pet Atheris squamigera. Edema was noted soon after presentation, with edema extending proximally throughout admission.

A 26-year-old-man without past medical history presented to the emergency department (ED) for evaluation of left foot pain and swelling caused by a snakebite. The patient handled multiple venomous snakes at home, which included an African viper species: *Atheris squamigera* (Figure 1). Earlier that day while attempting to transfer the snake between containers, he was bitten twice on the dorsal aspect of his left foot (Figure 2). Upon presentation to the ED, the patient’s vital signs included: blood pressure (BP) 112/80 mmHg, heart rate 77 beats per minute, temperature 98.5°F, respiratory rate 16 breaths per minute, and room air oxygen saturation 99%. Initial physical examination revealed small puncture wounds noted on the dorsum of the left foot with surrounding erythema, ecchymosis, and edema. Within two hours of presentation, the edema extended proximally to the left ankle. Abnormal lab values included an elevated D-dimer 1.06 (reference range <0.50 ug/mL), white blood cell count 12.58 (reference range 4.0-10.5 thou/cmm), and absolute neutrophil count 9.15 (reference range 1.8-7.8 thou/cmm). Normal laboratory testing included fibrinogen, prothrombin time/international normalized ratio, partial thromboplastin time, and creatinine. After consultation with a regional medical toxinologist, the patient was transferred to a tertiary care center for further management.

Upon arrival at the tertiary care hospital, a repeat examination of the left distal extremity identified persistent edema and tenderness in the foot (Figure 2). Repeat lab testing revealed a low fibrinogen 144 (reference range 180-500 mg/dL) and an elevated D-dimer greater than 20 (reference range <0.50 ug/mL). The medical toxicology team consulted with the physician and primary investigator of the Miami-Dade Fire Rescue Venom One team and developed a plan to obtain antivenom. The medical director and toxicologist of Venom One stated that, while there was no antivenom specifically for *Atheris squamigera*, an antivenom for other species could be beneficial [3]. Antivenom therapy was recommended if the patient demonstrated progressive systemic toxicity.

The patient’s symptoms remained local and mild and did not require analgesia. No systemic toxicity developed, and antivenom was not administered. He was discharged on hospital day two (HD2) and an outpatient follow-up was arranged. Abnormal lab results at the time of discharge included an improved D-dimer at 18.73 (reference range <0.50 ug/mL) and low fibrinogen of 121 (reference range 180-500 mg/dL), with all additional lab results being within normal limits.

## Discussion

*Atheris squamigera* is an arboreal, venomous snake species typically located in Western and Central Africa [4]. This species lives in the rainforests of the tropical sub-Saharan region. Currently, there are no specific antivenoms available for this species, but antivenoms for other species have been used in cases of severe envenomation [3].

Only rarely have *Atheris squamigera* envenomations been documented in the US literature, and, when reported, seem to be associated with exotic pet ownership [3,5]. In one case, the patient developed coagulopathy, and three doses of Near Middle East Antivenom were administered [3]. Near Middle East Antivenom combines antivenom of four snake species: *Echis, Naja, Cewrastes*, and *Vipera *[3]. The second US case described a patient who developed thrombocytopenia and hypofibrinogenemia, with normalization of labs and was discharged home by HD2. Platelets and cryoprecipitate were transfused, and the patient did not require antivenom [5]. In this case of African Bush Viper envenomation, the mentioned Near Middle East Antivenom would not be utilized as there are now newer, safe polyvalent antivenoms available that would have been recommended had his clinical condition deteriorated. With consideration of the clinical presentation and the rarity of this venomous snake in the US, the authors recommend consultation with a regional poison center, medical toxicologist, or toxinologist for these cases.

Occasionally, snake bites are “dry bites” in which no clinical envenomation occurs. Roughly 25% of viper bites do not cause envenomation [5]. An early indicator of possible envenomation is the observation of neutrophilic leukocytosis [6], which was evident in this patient. Moreover, the clinical examination findings of swelling, ecchymosis, and erythema, in addition to the mild lab abnormalities, indicated that this patient was envenomated. However, progressive limb swelling, coagulopathy, or hemorrhage, which may develop as early as two hours after a bite [3], did not occur.

Envenomation by non-native snake species in the US poses a significant challenge to physicians given both the lack of familiarity with the species and difficulty procuring exotic antivenom. Two previous US studies examined snakebites from non-native or exotic species. One of those studies surveyed Pennsylvania poison control centers' reports of exotic snakebites during a 14-year period [7]. Eighteen non-native, envenomated snakebite patients were identified, and in most cases, antivenom to treat these patients was difficult to obtain. It was also reported that since venomous, non-native snakebites are infrequent, available vials of antivenom had expired [7]. The second study examined envenomation across the US and found 258 snakebites from non-native species during a six-year period [8]. The authors discussed the increasing difficulty for hospitals to obtain antivenom due to various regulations. Typically, zoos are the only institutions that store antivenoms and will only supply antivenoms for the species in their collection. Both studies reported that it took extended periods of time to obtain the necessary antivenoms when needed.

## Conclusions

This case represents the third published case of *Atheris squamigera* envenomation in the US. Physicians must be aware of both the possibility of exotic snake bites and the process of procuring rare antivenoms if needed. The increase in practice of exotic pet ownership and sales makes this important, especially in regions where toxicology experts are not easily accessible. Consultation with a regional poison center, medical toxicologist, or toxinologist is recommended by the authors. Moreover, databases and antivenom supplies should be available and updated for emergency situations.
